# A new upper bound of geometric constant $D(X)$

**DOI:** 10.1186/s13660-017-1474-0

**Published:** 2017-08-31

**Authors:** Jin Huan Li, Bo Ling, San Yang Liu

**Affiliations:** School of Mathematics and Statistics, Xidian University, Taibai Road, Xi’an, 710071 P.R. China

**Keywords:** Birkhoff orthogonality, isosceles orthogonality, symmetric normed linear spaces, geometric constant

## Abstract

A new constant $\mathit{WD}(X)$ is introduced into any real $2^{n}$-dimensional symmetric normed space *X*. By virtue of this constant, an upper bound of the geometric constant $D(X)$, which is used to measure the difference between Birkhoff orthogonality and isosceles orthogonality, is obtained and further extended to an arbitrary *m*-dimensional symmetric normed linear space ($m\geq2$). As an application, the result is used to prove a special case for the reverse Hölder inequality.

## Introduction

The notion of orthogonality has many forms when the underlying space is transferred from inner product spaces to real normed spaces. For example, Birkhoff [1] introduced Birkhoff orthogonality in which *X* is assumed to be a real normed linear space. If $\Vert x+\lambda y \Vert \geq \Vert x \Vert $, $\forall\lambda\in \mathbb{R}$, then *x* is said to be Birkhoff orthogonal to *y*. It can be written as $x \perp_{B} y$. James [2] defined isosceles orthogonality, that is, if $\Vert x+y \Vert =\Vert x-y \Vert $, then *x* is said to be isosceles orthogonal to *y*. It is denoted by $x\perp_{I} y$. When *X* is an inner product space, these two types of orthogonality are equivalent to inner-product orthogonality.

However, these two types of orthogonality are different in general linear normed spaces. In order to quantify their difference in a real normed space *X*, Ji and Wu [3] introduced the geometric constant $D(X)$

$$ D(X)= \inf\Bigl\{ \inf_{\lambda\in\mathbb{R}}\Vert x+\lambda y \Vert : x,y \in S(X), x \perp_{I} y \Bigr\} , $$

where $S(X)$ is the unit sphere of *X*, and obtained the bounds $2(\sqrt {2}-1)\leq D(X)\leq1$. In particular, they provided the value of $D(X)$ in any two-dimensional symmetric Minkowski plane. Recently, the value of the constant $D(X)$ in the normed plane whose unit circle is affine regular (4*n*)-gon was given in [4], and a new lower bound $c_{B}(\cdot)$ of $D(\cdot)$ was obtained in [5]. Note that the constant $D(X)$ is considered only in the unit sphere $S(X)$. In reference [6], the author considered two constants $BI(X)$ and $\mathit{IB}(X)$ to measure the difference between Birkhoff orthogonality and isosceles orthogonality in the entire space *X*:

$$\begin{aligned}& \mathit{BI}(X)=\sup\biggl\{ \frac{\Vert x+y \Vert -\Vert x-y \Vert }{\Vert x \Vert }: x,y\in X, x,y\neq0, x \perp _{B} y \biggr\} \end{aligned}$$

and

$$\begin{aligned}& \mathit{IB}(X)=\sup\biggl\{ \frac{\inf_{\lambda\in\mathbb{R}}\Vert x+\lambda y \Vert }{\Vert x \Vert }: x,y\in X, x,y\neq0, x \perp _{I} y \biggr\} . \end{aligned}$$

And the estimations $0\leq \mathit{BI}(X)\leq2$ and $\frac{1}{2}\leq \mathit{IB}(X)\leq 1$ were also obtained. Other constants used to measure the difference between Birkhoff orthogonality and Robert orthogonality [7] were studied by [5] and [8]. For more conclusions about the difference between orthogonality types, please refer to literature of references [3–10] and so on.

In this study, by considering the constant $D(X)$ in $2^{n}$-dimensional real symmetric normed linear spaces, we obtain an upper bound $\mathit{WD}(X)$. As we discuss in Corollary 1, this bound can be extended to any *m*-dimensional symmetric normed linear space ($m\geq 2$). This article is organized as follows. In Section 2, we present some notations and definitions. In Section 3, the constant $\mathit{WD}(X)$ is introduced and discussed. In Section 4, we consider $\mathit{WD}(X)$ for the space $l_{p}^{2^{n}}$ and present a special case for the reverse Hölder inequality.

## Preliminaries

Let us fix some notations. Let *X* be an *n*-dimensional real linear normed space. By $\Vert \cdot \Vert $ and $\Vert \cdot \Vert ^{\ast}$, we denote the norm of *X* and the norm of a dual space $X^{\ast}$, respectively. The notation $S(X)$ is the unit sphere of *X*. Let $\mathbb{R}$ and $\mathbb{N}$ denote the real field and a positive integer set, respectively.

### Definition

Let *X* be an *n*-dimensional real normed linear space. If there exist $e_{1},e_{2}, \ldots, e_{n}\in S(X)$ such that, for any $a_{i}\in\mathbb{R}$, $i=1,2, \ldots, n$, the following equality

$$ \bigl\Vert \vert a_{1} \vert e_{1}+\vert a_{2} \vert e_{2}+\cdots+\vert a_{n} \vert e_{n} \bigr\Vert =\Vert a_{1}e_{1}+a_{2} e_{2}+\cdots+a_{n}e_{n} \Vert = \Biggl\Vert \sum_{k=1}^{n}\pm a_{\Phi(i)}e_{i} \Biggr\Vert $$

always holds, where $\Phi(i)\in\{1,2, \ldots,n\}$ and $\Phi(i)\neq \Phi(j)$ (if $i\neq j$), then we call *X* a symmetric normed linear space and $\{e_{1},e_{2}, \ldots, e_{n}\}$ a group of symmetric axes of *X*. In particular, we call $\sum_{k=1}^{n}\pm a_{\Phi(i)}e_{i}$ a symmetric element of *x*.

Let *X* be an *n*-dimensional symmetric normed linear space and $e_{1}, \ldots, e_{n}$ be a group of symmetric axes. For $x\in X$, *x* is denoted by the coordinate representation of this group of symmetric axes, i.e., $x=(x_{1},\ldots,x_{n})=x_{1}e_{1}+\cdots+x_{n}e_{n}$.

## Main results

Firstly, the following elementary results are presented. Throughout this paper, the symbol $\langle\cdot, \cdot\rangle$ denotes the natural inner product of two *n*-dimensional vectors. The first two lemmas are known, but we fail to find literature sources.

### Lemma 1

*Let* *X* *be a normed space* $(\mathbb{R}^{n}, \Vert \cdot \Vert )$ *and* $e_{1}=(1,0,\ldots, 0)$, $e_{2}=(0,1,0,\ldots, 0), \ldots, e_{n}=(0,0,\ldots, 0,1)$ *be a basis of X*. *Assume that* *B* *is a skew*-*symmetric matrix*, *i*.*e*., $B^{T}=-B$. *Then* $\langle x,Bx\rangle=0$ *for any* $x\in X$.

### Proof

Given that $\langle x,Bx\rangle=\langle B^{T}x,x\rangle=-\langle x,Bx\rangle$, then $\langle x,Bx\rangle=0$. □

### Lemma 2

*Let* *X* *be a normed space* $(\mathbb{R}^{2^{n}}, \Vert \cdot \Vert )$ *and* $e_{1}=(1,0,\ldots, 0)$, $e_{2}=(0,1,0,\ldots, 0), \ldots, e_{2^{n}}=(0,0,\ldots, 0,1)$ *be a basis of X*. *There exist rank* $2^{n}$ *matrices*

$$ B_{2^{n},1}, B_{2^{n},2}, \ldots, B_{2^{n},2^{n}-1} $$

*such that*

(i) *each square matrix* $B_{2^{n},i}$ ($i=1, 2, \ldots,2^{n}-1$) *is a skew*-*symmetric orthogonal matrix*, *i*.*e*., $B_{2^{n},i}^{T}=-B_{2^{n},i}$, $B_{2^{n},i}^{T}B_{2^{n},i}=Id_{2^{n}}$;
(ii) *each row and column in* $B_{2^{n},i}$ ($i=1, 2, \ldots, 2^{n}-1$) *has one and only one non*-*zero element*, *and this element is* 1 *or* −1;
(iii) *matrices* $B_{2^{n},i}^{T} B_{2^{n},j}$, $i\neq j$, *i*, $j=1, 2, \ldots, 2^{n}-1$, *satisfy the preceding two properties*, *i*.*e*., (i) *and* (ii).

### Proof

The result is proven by induction. For $n=1$, the only matrix

$$B_{2,1}= \begin{bmatrix} 0 & -1 \\ 1 & 0 \end{bmatrix} $$

satisfies conditions (i)-(iii).

Assume that this lemma holds for $n=k$, namely, the matrices $B_{2^{k},1}$, $B_{2^{k},2}, \dots, B_{2^{k},2^{k}-1}$ exist, satisfying the conditions (i) to (iii).

Now, we shall prove this lemma holds for $n=k+1$. First, we need to introduce three rank 2 square matrices $\sigma_{1}$, $\sigma_{2}$, *σ* and zero matrix **0**, which are defined as

$$ \sigma_{1}= \begin{bmatrix} 1 & 0 \\ 0 & -1 \end{bmatrix} , \qquad\sigma_{2}= \begin{bmatrix} 0 & 1 \\ 1 & 0 \end{bmatrix} , \qquad\sigma= \begin{bmatrix} 0 & -1 \\ 1 & 0 \end{bmatrix} , \qquad\mathbf{0} = \begin{bmatrix} 0 & 0 \\ 0 & 0 \end{bmatrix} , $$

respectively. Next, we claim that the set $\{B_{2^{k+1},1}, B_{2^{k+1},2}, \ldots, B_{2^{k+1},2^{k+1}-1}\}$ can be written as the following set:

1 $$ \bigl\{ B_{2^{k},i}(\sigma_{1}),B_{2^{k},i}( \sigma_{2}), Id_{2^{k}}(\sigma): i=1,2,\ldots ,2^{k}-1 \bigr\} . $$

So we only need to prove that the matrices in (1) satisfy the three properties in this lemma, where $B_{2^{k},i}(\sigma_{j})$ denotes the matrix in which the entries 1, −1, 0 in matrix $B_{2^{k},i}$ are replaced by the matrices $\sigma_{j}$, $-\sigma _{j}$, **0** respectively; and $Id_{2^{k}}(\sigma)$ denotes the matrix in which the entries 1, 0 in the unit matrix $Id_{2^{k}}$ are replaced by the matrices *σ*, **0**, respectively.

Let

$$ \bigl[ B_{2^{k},i}(\sigma_{j}) \bigr]^{T} B_{2^{k},i}(\sigma_{j}) =B_{2^{k},i}^{T} \bigl( \sigma_{j}^{T} \bigr)B_{2^{k},i}(\sigma _{j}) =Id_{2^{k}} \bigl( \sigma_{j}^{T} \sigma_{j} \bigr) =Id_{2^{k+1}}, $$

where $i=1, \dots, 2^{k}-1$, $j=1, 2$, and

$$ \bigl[Id_{2^{k}}(\sigma) \bigr]^{T} \mathit{Id}_{2^{k}}(\sigma )= \mathit{Id}_{2^{k}} \bigl(\sigma^{T} \bigr)\mathit{Id}_{2^{k}}(\sigma )= \mathit{Id}_{2^{k}} \bigl(\sigma^{T}\sigma\bigr)=\mathit{Id}_{2^{k+1}}. $$

Then the matrices in (1) are proven to be orthogonal.

By induction, $B_{2^{k},i}^{T} B_{2^{k},l}$ (where $i\neq l$, $j=1,2$) is a skew-symmetric orthogonal matrix and

$$ \bigl[ B_{2^{k},i}(\sigma_{j}) \bigr]^{T} B_{2^{k},l}(\sigma_{j}) = B_{2^{k},i}^{T} \bigl( \sigma_{j}^{T} \bigr) B_{2^{k},l}(\sigma _{j}) = B_{2^{k},i}^{T}B_{2^{k},l} \bigl(\sigma _{j}^{T} \sigma_{j} \bigr)=B_{2^{k},i}^{T} B_{2^{k},l}(\mathit{Id}_{2}). $$

We obtain that $[B_{2^{k},i}(\sigma_{j})]^{T} B_{2^{k},l}(\sigma _{j})$ is also a skew-symmetric orthogonal matrix that satisfies condition (ii). Similarly, $[B_{2^{k},i}(\sigma_{j})]^{T} B_{2^{k},l}(\sigma_{m})$, $i\neq l$, $j\neq m$ and $[B_{2^{k},i}(\sigma_{j})]^{T} \mathit{Id}_{2^{k}}(\sigma) $ satisfy conditions (i) and (ii). Thus, the matrices in set (1) satisfy condition (iii). □

In order to present an upper bound of $D(X)$, a new constant $\mathit{WD}(X)$ for any real normed linear space $X=(\mathbb{R}^{2^{n}}, \Vert \cdot \Vert )$ is introduced.

### Definition 1

Let *X* be a normed space $(\mathbb{R}^{2^{n}}, \Vert \cdot \Vert )$ and $e_{1}=(1,0,\ldots, 0)$, $e_{2}=(0,1,0,\ldots, 0), \ldots, e_{2^{n}}=(0,0,\ldots, 0,1)$ be a basis of *X*. The geometric constant $\mathit{WD}(X)$ is defined as

$$ \mathit{WD}(X)=\inf\Bigl\{ \inf_{\lambda\in\mathbb{R}}\Vert x+\lambda y \Vert : x \in S(X), y=Bx, B\in\{B_{2^{n},1}, B_{2^{n},2}, \ldots, B_{2^{n},2^{n}-1}\} \Bigr\} , $$

where $B_{2^{n},1}$, $B_{2^{n},2}, \dots, B_{2^{n},2^{n}-1}$ are given as in Lemma 2.

### Proposition 1

*Let* *X* *be a normed space* $(\mathbb{R}^{2^{n}}, \Vert \cdot \Vert )$ *and* $e_{1}=(1,0,\ldots, 0)$, $e_{2}=(0,1,0,\ldots, 0), \dots, e_{2^{n}}=(0,0,\ldots, 0,1)$ *be a basis of X*. *And the normed space* *X* *is such that*, *for any* $x\in S(X)$ *and any* $y=B_{2^{n},i}x$, $B_{2^{n},i}\in\{B_{2^{n},1}, \ldots, B_{2^{n},2^{n}-1}\}$, *there exists* $\lambda_{0}\in\mathbb{R}$ *such that* $x+\lambda_{0} y \perp _{B} H$, *where* $H=\operatorname{span}\{y, B_{2^{n},1}x, \ldots, B_{2^{n},i-1}x, B_{2^{n},i+1}x,\ldots, B_{2^{n},2^{n}-1}x\}$. *Then*

2 $$ \mathit{WD}(X) = \min\biggl\{ \frac{\langle x,x\rangle}{ \Vert x \Vert ^{\ast}}: x \in S(X) \biggr\} =\min \biggl\{ \frac{\langle x,x\rangle}{ \Vert x \Vert \Vert x \Vert ^{\ast}}: x\neq0, x \in X \biggr\} . $$

### Proof

Assume that $x \in S(X)$, $y=Bx$, where $B\in\{ B_{2^{n},1}, B_{2^{n},2}, \dots, B_{2^{n},2^{n}-1}\}$. Without losing generality, let $y=B_{2^{n},1}x$. *Then there exists* $\lambda_{0}\in\mathbb{R}$ such that $\Vert x +\lambda_{0}y \Vert = \min_{\lambda\in\mathbb{R}} \Vert x +\lambda y \Vert $, i.e., $x+\lambda_{0} y \perp_{B} y$. Based on Corollary 4.2 in [11], we have the following equalities:

$$ f_{1}(y)= 0, \qquad f_{1}(x+\lambda_{0} y) = \Vert x+\lambda_{0} y \Vert , \qquad \Vert f_{1} \Vert ^{\ast} =1, $$

for some $f_{1} \in X^{\ast}$. Let $M_{y}=\{f\in X^{\ast}: f(y) = 0\} $. Then $\operatorname{dim}(M_{y})=2^{n}-1$. Based on Lemma 2, the matrices $B_{2^{n},i}$ and $B_{2^{n},i}^{T}B_{2^{n},j}$ ($i\neq j$) are skew-symmetric. Then, by Lemma 1, the following can be obtained:

$$ \langle x,B_{2^{n},i}x\rangle=0, \qquad\langle B_{2^{n},i}x,B_{2^{n},j}x \rangle=0, \quad i\neq j, $$

which imply that all of the vectors *x*, $B_{2^{n},2}x, \dots, B_{2^{n},2^{n}-1}x$ are in $M_{y}$ and linearly independent. Thus,

$$ M_{y}=\operatorname{span}\{x, B_{2^{n},2}x, \dots, B_{2^{n},2^{n}-1}x\} \bigl( \subset X^{\ast} \bigr). $$

Given that $f_{1}\in M_{y}$, we may assume that $f_{1}=\alpha_{1}x +\alpha_{2} B_{2^{n},2}x+\cdots+\alpha_{2^{n}-1}B_{2^{n},2^{n}-1}x$, where $\alpha_{1}, \ldots, \alpha_{2^{n}-1}\in\mathbb{R}$. We get $f_{1}(x+\lambda_{0} y)=\langle\alpha_{1}x, x\rangle=\langle\alpha _{1}x, x+\lambda_{0} y\rangle=\Vert x+\lambda_{0} y \Vert $. This leads to $f_{1}\vert _{\operatorname{span}\{x, y\}}=\alpha_{1}x \vert _{\operatorname{span}\{x, y\}}$ and $\Vert \alpha_{1}x\vert _{\operatorname{span}\{x, y\}} \Vert ^{\ast}\geq1$. Hence, we obtain that $1\leq \Vert f_{1} \vert _{\operatorname{span}\{x, y\}} \Vert ^{\ast}\leq \Vert f_{1} \Vert ^{\ast}=1$. Then we have $\Vert \alpha_{1}x\vert_{\operatorname{span}\{x, y\}} \Vert ^{\ast}=1$.

Since $x+\lambda_{0} y\perp_{B}H$, where $H=\operatorname{span}\{y, B_{2^{n},2}x, \ldots, B_{2^{n},2^{n}-1}x\}\subset X$, and $x\notin H$, then $X=\operatorname{span}\{ x+\lambda_{0} y\}+H$. Thus, for any $z\in H$ and any real number *a*, *b*, the following inequality

$$ \bigl\Vert a(x+\lambda_{0} y) \bigr\Vert \leq\bigl\Vert a(x+ \lambda_{0} y)+bz \bigr\Vert $$

holds. Thus, the inequalities $\vert \langle\alpha_{1}x, a(x+\lambda_{0} y)+bz\rangle \vert \leq \Vert \alpha_{1}x\vert _{\operatorname{span}\{x, y\}} \Vert ^{\ast} \Vert a(x+\lambda_{0} y) \Vert \leq \Vert a(x+\lambda_{0} y)+bz \Vert $ indicate that $\Vert \alpha_{1}x \Vert ^{\ast}\leq1$, and then $\Vert \alpha_{1}x \Vert ^{\ast}=1$, namely, $\vert \alpha_{1} \vert =\frac {1}{\Vert x \Vert ^{\ast}}$ is independent of *y* and $\Vert x+\lambda_{0} y \Vert =\langle\alpha_{1}x, x\rangle$. So Eq. (2) is obtained, and thereby we complete the proof. □

### Lemma 3

*Let* *X* *be a real symmetric linear normed space* $(\mathbb{R}^{2^{n}}, \Vert \cdot \Vert )$ *and* $e_{1}=(1,0,\ldots, 0)$, $e_{2}=(0,1,0,\ldots, 0), \dots, e_{2^{n}}=(0,0,\ldots, 0,1)$ *be a basis of X*.

1. *Assume that each row and each column in the* $2^{n}\times 2^{n}$ *matrix* *B* *has only one non*-*zero element*, *which takes the value of* 1 *or* −1. *Then matrix* *B* *is an isometric operator on X*.
2. *Assume that*
  (i) *B* *is a skew*-*symmetric and orthogonal matrix*, *i*.*e*., $B^{T}=-B$, $B^{T}B=Id$;
  (ii) *Each row and each column in matrix* *B* *has only one non*-*zero element*, *which takes the value of* 1 *or* −1.
  *Then* $\Vert Bx \Vert =\Vert x \Vert $ *and* $x\perp_{I} Bx$ *for any* $x\in X$.

### Proof

(1) The equality $y=Bx$ indicates that *y* is merely the vector in which the elements are a rearrangement of the corresponding elements of *x*; some items in the elements change their sign. Thus, based on the definition of a real symmetric linear normed space, we have $\Vert Bx \Vert =\Vert x \Vert $.

(2) Let $y=Bx$, by Lemma 3(1), *B* is an isometric operator. Thus, $\Vert Bx \Vert =\Vert x \Vert =1$. Meanwhile, $\Vert x+y \Vert =\Vert (Id+B)x \Vert =\Vert B(Id-B)x \Vert =\Vert (Id-B)x \Vert =\Vert x-y \Vert $, and $x \perp_{I} y$ are obtained. □

Then the main theorem can be obtained.

### Theorem 1

*Let* *X* *be a real symmetric linear normed space* $(\mathbb{R}^{2^{n}}, \Vert \cdot \Vert )$ *and* $e_{1}=(1,0,\ldots, 0)$, $e_{2}=(0,1,0,\ldots, 0), \dots, e_{2^{n}}=(0,0,\ldots, 0,1)$ *be a basis of X*. *And the normed space* *X* *is such that*, *for any* $x\in S(X)$ *and any* $y=B_{2^{n},i}x$, $B_{2^{n},i}\in\{B_{2^{n},1}, \ldots, B_{2^{n},2^{n}-1}\}$, *there exists* $\lambda_{0}\in\mathbb{R}$ *such that* $x+\lambda_{0} y \perp_{B} H$, *where* $H=\operatorname{span}\{y, B_{2^{n},1}x, \ldots, B_{2^{n},i-1}x, B_{2^{n},i+1}x,\ldots, B_{2^{n},2^{n}-1}x\}$. *Then* $2(\sqrt{2}-1 )\leq D(X) \leq \mathit{WD}(X)\leq1$.

### Proof

The first inequality has been proven in Theorem 1 of [3]; thus, the last inequality can be easily obtained. The second inequality can be proven as follows by assuming that $x\in S(X)$. Given that *X* is a symmetric normed linear space and $B_{2^{n},i}$, $i=1,\ldots, 2^{n}-1$, satisfies properties (i) and (ii) in Lemma 2. By Lemma 3(2), $B_{2^{n},i} x \in S(X)$ and $x\perp _{I} B_{2^{n},i}x$ ($i=1, \ldots, 2^{n}-1$) can be obtained. Hence, we get $D(X) \leq \mathit{WD}(X)$. □

It is easy to extend the above result to any *m*-dimensional real symmetric normed linear space.

### Corollary 1

*Let* *X* *be a real symmetric linear normed space* $(\mathbb{R}^{m}, \Vert \cdot \Vert )$ *and* $e_{1}=(1,0,\ldots, 0)$, $e_{2}=(0,1,0,\ldots, 0), \dots, e_{m}=(0,0,\ldots, 0,1)$ *be a basis of X*. *And the normed space* *X* *is such that there exists a subspace* $Y\subset X$ *with* $\operatorname{dim} Y=2^{n}$ *and*, *for any* $x\in S(Y)$ *and any* $y=B_{2^{n},i}x$, $B_{2^{n},i}\in\{B_{2^{n},1}, \ldots, B_{2^{n},2^{n}-1}\}$, *there exists* $\lambda_{0}\in\mathbb{R}$ *such that* $x+\lambda_{0} y \perp_{B} H$, *where* $H=\operatorname{span}\{y, B_{2^{n},1}x, \ldots, B_{2^{n},i-1}x, B_{2^{n},i+1}x,\ldots, B_{2^{n},2^{n}-1}x\}$. *Then* $2(\sqrt{2}-1 )\leq D(X) \leq \mathit{WD}(Y)\leq1$.

It is worth mentioning that the upper bound $\mathit{WD}(X)$ of the geometric constant $D(X)$, which is given in Theorem 1, has several advantages. Firstly, it is defined unrelated to isosceles orthogonality compared to $D(X)$. Secondly, due to (2), $\mathit{WD}(X)$ has a simple expression, which makes calculation feasible. Finally, it is less than one in general. For example, we consider $\mathit{WD}(X)$ for the space $l_{p}^{2^{n}}$ in the next section.

## The case of $l_{p}^{2^{n}}$

The space $l_{p}^{2^{n}}$ is used to show that the aforementioned upper bound $\mathit{WD}(X)$ is optimal for $D(X)$.

### Proposition 2

*Let* $p, q\geq1$ *such that* $\frac {1}{p}+\frac{1}{q}=1$. *Then*

$$\begin{aligned} \mathit{WD} \bigl(l_{p}^{2^{n}} \bigr)= \inf\biggl\{ \frac {1+t^{2}}{(1+t^{q})^{\frac{1}{q}}(1+t^{p})^{\frac{1}{p}}}: t \in[0,1] \biggr\} . \end{aligned}$$

### Proof

Assume that $x= \alpha_{1} e_{1} +\alpha_{2} e_{2}+\cdots + \alpha_{2^{n}} e_{2^{n}}\in S(l_{p}^{2^{n}})$ and $y=B_{2^{n},i}x,B_{2^{n},i}\in\{B_{2^{n},1}, \ldots, B_{2^{n},2^{n}-1}\}$. For simplicity, we may take $\alpha_{i} \geq0$, $i=1,2,\ldots, 2^{n}$, and

$$\begin{aligned} y&= \sum_{k=1}^{2^{n-2}} \alpha_{2^{n-1}-k+1} e_{k}+\sum_{k=1}^{2^{n-2}} (-1) \alpha _{2^{n-2}-k+1} e_{2^{n-2}+k} \\ &\quad {}+ \sum_{k=1}^{2^{n-2}} \alpha_{2^{n}-k+1} e_{2^{n-1}+k}+\sum_{k=1}^{2^{n-2}} (-1) \alpha _{2^{n-1}+2^{n-2}-k+1} e_{2^{n-1}+2^{n-2}+k}. \end{aligned}$$

Let $f(\lambda)=\Vert x+\lambda y \Vert ^{p}$ and

$$\begin{aligned} f_{1}(\lambda)&=\sum_{k=1}^{2^{n-2}} (\alpha_{k}+\lambda\alpha_{2^{n-1}-k+1})^{p} +\sum _{k=1}^{2^{n-2}}(\alpha_{2^{n-2}+k}-\lambda \alpha_{2^{n-2}-k+1})^{p} \\ &\quad {}+\sum_{k=1}^{2^{n-2}} ( \alpha _{2^{n-1}+k}+\lambda\alpha_{2^{n}-k+1} )^{p} +\sum _{k=1}^{2^{n-2}} (\alpha_{2^{n-1}+2^{n-2}+k}-\lambda\alpha _{2^{n-1}+2^{n-2}-k+1})^{p}. \end{aligned}$$

Then the equality $f_{1}(\lambda)=f(\lambda)$ holds on the interval $[\xi,\eta]$, where

$$ \xi=\max\biggl\{ \max_{k=1}^{2^{n-2}} \biggl\{ - \frac{\alpha_{k}}{\alpha_{2^{n-1}-k+1}} \biggr\} , \max _{k=1}^{2^{n-2}} \biggl\{ - \frac{\alpha_{2^{n-1}+k}}{\alpha_{2^{n}-k+1}} \biggr\} \biggr\} $$

and

$$ \eta=\min\biggl\{ \min_{k=1}^{2^{n-2}} \biggl\{ \frac{\alpha_{2^{n-2}+k}}{\alpha_{2^{n-2}-k+1}} \biggr\} , \min _{k=1}^{2^{n-2}} \biggl\{ \frac{\alpha_{2^{n-1}+2^{n-2}+k}}{\alpha_{2^{n-1}+2^{n-2}-k+1}} \biggr\} \biggr\} . $$

Since $f(\lambda)$ is a convex function on $[\xi,\eta]$, then $f_{1}(\lambda)$ is also a convex function on $[\xi,\eta]$. There exists one case in which the following equalities about *λ* hold:

$$\begin{aligned}& \alpha_{2^{n-1}}(\alpha_{1}+\lambda\alpha _{2^{n-1}})^{p-1}= \alpha_{1} (\alpha_{2^{n-2}+2^{n-2}}- \lambda\alpha_{1})^{p-1}, \\& \alpha_{2^{n-1}-1}(\alpha_{2}+\lambda\alpha_{2^{n-1}-1})^{p-1}= \alpha_{2} (\alpha_{2^{n-1}-1}-\lambda\alpha_{2})^{p-1}, \\& \ldots, \\& \alpha_{2^{n-2}+1}(\alpha_{2^{n-2}}+\lambda\alpha _{2^{n-2}+1})^{p-1}= \alpha_{2^{n-2}}(\alpha_{2^{n-2}+1}- \lambda\alpha_{2^{n-2}})^{p-1}, \\& \ldots, \\& \alpha_{2^{n}}( \alpha_{2^{n-1}+1}+\lambda\alpha_{2^{n}} )^{p-1}= \alpha_{2^{n-1}+1}(\alpha_{2^{n}}-\lambda\alpha _{2^{n-1}+1})^{p-1}, \\& \ldots, \\& \alpha_{2^{n}-2^{n-2}+1}( \alpha_{2^{n-1}+2^{n-2}}+\lambda\alpha _{2^{n}-2^{n-2}+1} )^{p-1}=\alpha_{2^{n-1}+2^{n-2}}(\alpha_{2^{n-1}+2^{n-2}+1}-\lambda\alpha _{2^{n-1}+2^{n-2}})^{p-1}. \end{aligned}$$

In this case, $f'_{1}(\lambda)=0$. If we let $\gamma_{k}=(\frac {\alpha_{2^{n-1}-k+1}}{\alpha_{k}})^{\frac{1}{p-1}}$, $1\leq k\leq 2^{n-2}$, then

$$ \lambda=\frac{\alpha_{2^{n-1}-k+1}-\gamma_{k}\alpha_{k}}{\alpha _{k}+\gamma_{k}\alpha_{2^{n-1}-k+1}}. $$

If we let $\gamma_{2^{n-2}+k}=(\frac{\alpha_{2^{n}-k+1}}{\alpha _{2^{n-1}+k}})^{\frac{1}{p-1}}$, $1\leq k\leq2^{n-2}$, then

$$ \lambda=\frac{\alpha_{2^{n}-k+1}-\gamma_{k}\alpha _{2^{n-1}+k}}{\alpha_{2^{n-1}+k}+\gamma_{k}\alpha_{2^{n}-k+1}}. $$

On the one hand, we have

$$ \lambda=\frac{\alpha_{2^{n-1}-k+1}-\gamma_{k}\alpha_{k}}{\alpha _{k}+\gamma_{k}\alpha_{2^{n-1}-k+1}}\leq\frac{\alpha _{2^{n-1}-k+1}}{\alpha_{k}},\quad1\leq k\leq 2^{n-2} $$

and

$$ \lambda=\frac{\alpha_{2^{n}-k+1}-\gamma_{k}\alpha _{2^{n-1}+k}}{\alpha_{2^{n-1}+k}+\gamma_{k}\alpha_{2^{n}-k+1}}\leq \frac{\alpha_{2^{n}-k+1}}{\alpha_{2^{n-1}+k}},\quad1\leq k\leq 2^{n-2}. $$

On the other hand, we have

$$ \lambda- \biggl(-\frac{\alpha_{k}}{\alpha_{2^{n-1}-k+1}} \biggr)=\frac{\alpha_{k}^{2}+\alpha_{2^{n-1}-k+1}^{2}}{\alpha _{2^{n-1}-k+1}(\alpha_{k}+\gamma_{k}\alpha_{2^{n-1}-k+1})}\geq 0,\quad1 \leq k\leq2^{n-2}, $$

and

$$ \lambda- \biggl(-\frac{\alpha_{2^{n-1}+k}}{\alpha_{2^{n}-k+1}} \biggr)=\frac{\alpha_{2^{n-1}+k}^{2}+\alpha_{2^{n}-k+1}^{2}}{\alpha _{2^{n}-k+1}(\alpha_{2^{n-1}+k}+\gamma_{k}\alpha_{2^{n}-k+1})}\geq 0,\quad1 \leq k\leq2^{n-2}. $$

These inequalities show that $\lambda\in[\xi,\eta]$. Hence, if all $\gamma_{k}$ and $\gamma_{2^{n-2}+k}$ ($1\leq k\leq2^{n-2}$) are equal, then the preceding equalities about *λ* are equal. If $f_{1}(\lambda)$ has a minimum value on the interval $[\xi,\eta]$, then $f(\lambda)$ also has a minimum value on the interval $[\xi,\eta]$.

Let $t_{k}=\frac{\alpha_{2^{n-1}-k+1}}{\alpha_{k}}$ and $t_{2^{n-2}+k}=\frac{\alpha_{2^{n}-k+1}}{\alpha_{2^{n-1}+k}}$ ($1\leq k\leq2^{n-2}$). If $\alpha_{1}=\cdots=\alpha_{2^{n-2}}=\alpha _{2^{n-1}+1}=\cdots=\alpha_{2^{n-1}+2^{n-2}}$ and $\alpha _{2^{n-2}+1}=\cdots=\alpha_{2^{n-1}}=\alpha_{2^{n}-2^{n-2}+1}=\cdots =\alpha_{2^{n}}$, then $t_{1}=t_{2}=\cdots=t_{2^{n-1}}$ are obtained. We may assume that $t_{1}=t_{2}=\cdots=t_{2^{n-1}}=t$, and take $\lambda_{0}=\frac {t-t^{q-1}}{1+t^{q}}$, where *q* is any positive number such that $\frac {1}{p}+\frac{1}{q}=1$, then $f'_{1}(\lambda_{0})=0$ and

$$\begin{aligned} f_{1}(\lambda_{0}) &= \alpha^{p}_{1} \bigl[(1+\lambda_{0} t)^{p}+(t-\lambda _{0})^{p} \bigr]+\alpha^{p}_{2} \bigl[(1+\lambda_{0} t)^{p}+(t-\lambda_{0})^{p} \bigr] \\ &\quad{}+ \cdots + \alpha^{p}_{2^{n-2}} \bigl[(1+\lambda_{0} t)^{p}+(t-\lambda_{0})^{p} \bigr]+\alpha ^{p}_{2^{n-1}+1} \bigl[(1+\lambda_{0} t)^{p}+(t-\lambda_{0})^{p} \bigr] \\ &\quad {}+ \cdots+\alpha^{p}_{2^{n-1}+2^{n-2}} \bigl[(1+\lambda _{0} t)^{p}+(t-\lambda_{0})^{p} \bigr] \\ &= \frac{(1+t^{2})^{p}}{(1+t^{q})^{p-1}(1+t^{p})}. \end{aligned}$$

Moreover, we may assume that $\alpha_{1}\geq\alpha_{2^{n-1}}$, then $0\leq t\leq1$. If $\alpha_{1}\leq\alpha_{2^{n-1}}$, then we can take $t=t_{k}=\frac{\alpha_{k}}{\alpha_{2^{n-1}-k+1}}$. The sufficient and necessary condition for the extreme points of a derived convex function is that it must be the stagnation point. Since $f_{1}(\lambda)$ is a strictly convex function, then $\lambda_{0}$ is unique. Hence, we have

$$\begin{aligned} \mathit{WD} \bigl(l_{p}^{2^{n}} \bigr) =&\inf\biggl\{ \inf\biggl\{ \frac{1+t_{1}^{2}}{(1+t_{1}^{q})^{\frac{1}{q}}(1+t_{1}^{p})^{\frac {1}{p}}}: t_{1}\in[0,1] \biggr\} , \ldots, \\ &\inf\biggl\{ \frac{1+t_{2}^{2}}{(1+t_{2}^{q})^{\frac {1}{q}}(1+t_{2}^{p})^{\frac{1}{p}}}:t_{2}\in[0,1] \biggr\} , \\ & \inf\biggl\{ \frac{1+t_{2^{n}-1}^{2}}{(1+t_{2^{n}-1}^{q})^{\frac {1}{q}}(1+t_{2^{n}-1}^{p})^{\frac{1}{p}}}:t_{2^{n}-1}\in[0,1] \biggr\} \biggr\} \\ =& \inf\biggl\{ \frac{1+t^{2}}{(1+t^{q})^{\frac{1}{q}}(1+t^{p})^{\frac {1}{p}}}: t\in[0,1] \biggr\} . \end{aligned}$$

 □

### Remark 1

According to the above proof, $\mathit{WD}(l_{p}^{2^{n}})$ is independent of the selection of *B*. Thus, it may verify the existence of the space *X* satisfying the condition of Proposition 1.

### Corollary 2

*Let* *m*, *n* *be any positive integer such that* $2^{n}\leq m$. *Then*

$$ 2(\sqrt{2}-1 )\leq D \bigl(l_{p}^{m} \bigr)\leq D \bigl(l_{p}^{2^{n}} \bigr)\leq \mathit{WD} \bigl(l_{p}^{2^{n}} \bigr)\leq1. $$

### Corollary 3

$\lim_{p\rightarrow\infty}D(l_{p}^{m})=2(\sqrt{2}-1 )$. *Specially*, $\mathit{WD}(l_{1}^{2^{n}})=2(\sqrt{2}-1 )$.

### Corollary 4

*Let* $p, q\geq1$ *such that* $\frac{1}{p}+\frac{1}{q}=1$. *Assume that* $l_{p}^{2^{n}}$ *has the property that*, *for any* $x\in S(l_{p}^{2^{n}})$ *and any* $y=B_{2^{n},i}x$, $B_{2^{n},i}\in\{B_{2^{n},1}, \ldots, B_{2^{n},2^{n}-1}\}$, *there exists* $\lambda_{0}\in\mathbb{R}$ *such that* $x+\lambda_{0} y \perp_{B} H$, *where*

$$ H=\operatorname{span}\{y, B_{2^{n},1}x, \ldots, B_{2^{n},i-1}x, B_{2^{n},i+1}x, \ldots, B_{2^{n},2^{n}-1}x\}. $$

*Then*, *for any* $x\in l_{p}^{2^{n}}$ *and* $x\neq0$, *there exists a positive constant* $b=\mathit{WD}(l_{p}^{2^{n}})$ *such that*

3 $$ 2(\sqrt{2}-1 )\Vert x \Vert _{p}\Vert x \Vert _{q}\leq b \Vert x \Vert _{p}\Vert x \Vert _{q}\leq\langle x,x\rangle\leq \Vert x \Vert _{p} \Vert x \Vert _{q}. $$

The third inequality in (3) is the classical Hölder inequality

$$ \sum_{k=1}^{n}a_{k}b_{k} \leq\Biggl(\sum_{k=1}^{n}a^{p}_{k} \Biggr)^{\frac{1}{p}} \Biggl(\sum_{k=1}^{n}b^{q}_{k} \Biggr)^{\frac{1}{q}}, $$

where $a_{k}\geq0$, $b_{k}\geq0$ ($k=1, 2, \dots, n$), $p>1$, $q>1$ and $\frac{1}{p}+\frac{1}{q}=1$. This classical inequality plays a very important role in many areas of pure and applied mathematics. Various generalizations and improvements of this classical inequality have been studied by many mathematicians. The second inequality in (3) is a special case of the reverse version of Hölder inequality, which differs from other known results (e.g., see [12]). One of its values is that the constant *b* is always greater than or equal to $2(\sqrt{2}-1 )$.

## Conclusion

In this paper, by studying the geometric constant $D(X)$ of any real $2^{n}$-dimensional symmetric normed space $X=(\mathbb{R}^{2^{n}}, \Vert \cdot \Vert )$, we obtained an upper bound $\mathit{WD}(X)$, which is not greater than 1. And using the special properties of a finite dimensional normed space $(\mathbb{R}^{2^{n}}, \Vert \cdot \Vert )$ and the constraints on $(\mathbb{R}^{2^{n}}, \Vert \cdot \Vert )$, we also give a simple formula for $\mathit{WD}(X)$. In particular, when $X = l_{p}^{2^{n}}$, this formula is used to give a special form of the reverse Hölder inequality.
